# Supplementary material for: Broadband sound barriers with bianisotropic metasurfaces
Source: Nat Commun. 2018 Dec 13;9:5299. doi: 10.1038/s41467-018-07809-3 (PMC6294143; doi:10.1038/s41467-018-07809-3)
Supplement: Supplementary file 1 — Supplementary Information [file 41467_2018_7809_MOESM1_ESM.pdf]

Supplementary material for the paper

"Broadband Sound Barriers with Bianisotropic Metasurfaces"

Popa et al.

Supplementary Note 1: Transmission Through Bianisotropic Metasurface

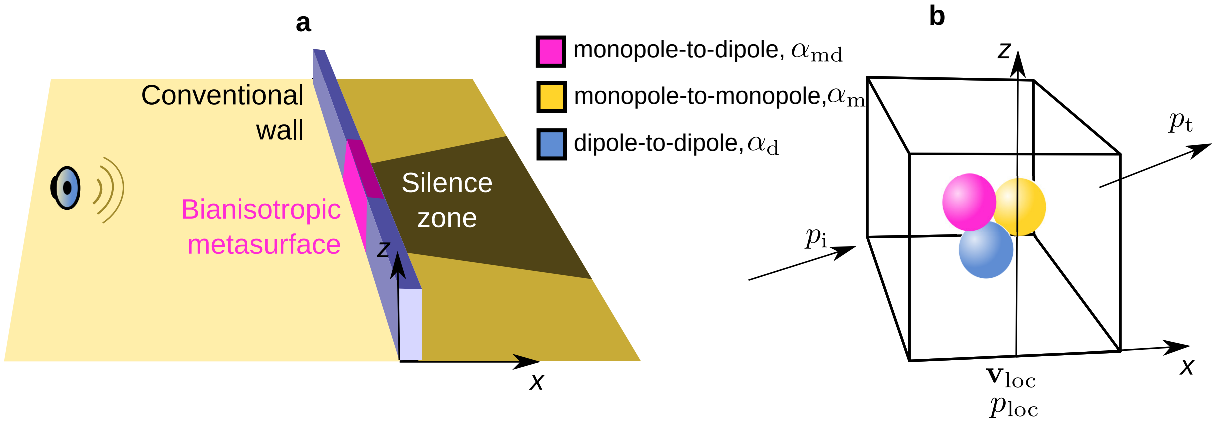

Supplementary Figure 1: Bianisotropic metasurface sound barrier. **a**, A section of a conventional wall shown in black is replaced by a bianisotropic metasurface window designed to block sound propagation behind the metasurface. **b**, The metasurface unit cell (meta-atom) is made of three types of sources whose acoustic responses are described in terms of bianisotropic polarizability $\boldsymbol{\alpha}_{\mathrm{md}}$ and conventional polarizabilities α_{m} and $\overline{\overline{\boldsymbol{\alpha}}}_{\mathrm{d}}$. The local pressure and particle velocity at the position of the sources are p_{loc} and $\mathbf{v}_{\mathrm{loc}}$.

Consider a sound source placed in front of the conventional wall shown in Supplementary Fig. 1a. A window is cut in the wall and replaced with a bianisotropic metasurface composed of meta-atoms whose functional behavior is summarized in Supplementary Fig. 1b. The meta-atom response to the incident wave p_{i} is described in terms of polarizabilities following a standard procedure [1–4]. Specifically, the meta-atom is modeled as a set of sources represented as spheres placed in a background fluid of characteristic impedance Z_0. Each source responds to either the monopole moment associated to the local pressure p_{loc} or the local particle velocity $\mathbf{v}_{\mathrm{loc}}$ associated to the dipole moment produced by the incident wave. In response to p_{loc} and $\mathbf{v}_{\mathrm{loc}}$ the sources generate either a monopole moment (non-zero local pressure field) or a dipole moment (non-zero local particle velocity).

The bianisotropic meta-atom presented here is composed of three sources that account for the conventional monopole-to-monopole, dipole-to-dipole, and bianisotropic monopole-to-dipole couplings. We assume a zero dipole-to-monopole coupling, thus breaking the reciprocity conditions in unbiased passive media [4–7]. We further assume that the conventional polarizabilities α_{m}, $\overline{\overline{\boldsymbol{\alpha}}}_{\mathrm{d}} = \alpha_{\mathrm{d}}\overline{\overline{\mathbf{I}}}$ are isotropic and the bianisotropic polarizability is $\boldsymbol{\alpha}_{\mathrm{md}} = \alpha_{\mathrm{md}}\hat{\mathbf{x}}$, where $\overline{\overline{\mathbf{I}}}$ is the second order unit tensor and $\hat{\mathbf{x}}$ is the unit vector pointing in the direction perpendiculat on the metasurface, as illustrated in the Supplementary Fig. 1a. Our goal is to find the pressure field transmitted through the metasurface p_{t} in terms of α_{m}, α_{d}, α_{md}, and the incident wave p_{i}.

The local fields produced by the three types of polarizabilities are given by

$$\begin{aligned} p_{\mathrm{m}} &= \alpha_{\mathrm{m}} p_{\mathrm{loc}}, \\ \mathbf{v}_{\mathrm{d}} &= \alpha_{\mathrm{d}} \mathbf{v}_{\mathrm{loc}}, \\ \mathbf{v}_{\mathrm{md}} &= \hat{\mathbf{x}} \alpha_{\mathrm{md}} Z_0^{-1} p_{\mathrm{loc}}. \end{aligned} \tag{1}$$

The dipole-to-dipole and monopole-to-dipole inclusions produce pure dipole moments (net particle velocity but zero local pressure), and the monopole-to-monopole inclusion produces pure monopole moments (net pressure but zero local particle velocity), therefore the local pressure and particle velocity are written

$$\begin{aligned} p_{\mathrm{loc}} &= p_{\mathrm{i}} + p_{\mathrm{m}}, \\ \mathbf{v}_{\mathrm{loc}} &= \mathbf{v}_{\mathrm{i}} + \mathbf{v}_{\mathrm{d}} + \mathbf{v}_{\mathrm{md}}. \end{aligned} \tag{2}$$

Equations (1) and (2) lead to the following local field expressions

$$\begin{aligned} p_{\mathrm{loc}} &= \frac{1}{1-\alpha_{\mathrm{m}}} p_{\mathrm{i}}, \\ \mathbf{v}_{\mathrm{loc}} &= \frac{1}{1-\alpha_{\mathrm{d}}} \mathbf{v}_{\mathrm{i}} + \hat{\mathbf{x}} \frac{\alpha_{\mathrm{md}}}{(1-\alpha_{\mathrm{m}})(1-\alpha_{\mathrm{d}})} \frac{p_{\mathrm{i}}}{Z_0}, \end{aligned} \tag{3}$$

where $\mathbf{v}_{\mathrm{i}}$ is the particle velocity associated with the incident wave.

The total pressure travelling through the metasurface is the superposition of the incident field

$p_{\rm i}$ and the fields scattered by the three inclusions

$$p_{\rm t} = p_{\rm i} + p_{\rm m} + p_{\rm d} + p_{\rm md}, \tag{4}$$

where the transmitted pressure fields propagating in the positive $\hat{\mathbf{x}}$ direction produced by the sources are obtained from equations (1) and (3) and are given by, respectively

$$\begin{aligned} p_{\rm m} &= \frac{\alpha_{\rm m}}{1-\alpha_{\rm m}} p_{\rm i}, \\ p_{\rm d} &= \frac{\alpha_{\rm d}}{1-\alpha_{\rm d}} p_{\rm i} + \frac{\alpha_{\rm d}\alpha_{\rm md}}{(1-\alpha_{\rm m})(1-\alpha_{\rm d})} p_{\rm i}, \\ p_{\rm md} &= \frac{\alpha_{\rm md}}{1-\alpha_{\rm m}} p_{\rm i}. \end{aligned} \tag{5}$$

In the above equations we took into account that the ratio pressure-to-particle-velocity is the characetristic impedance of the background fluid Z_0. Plugging the above expressions in equation (4) leads to the following expression of the transmitted pressure in the positive $\hat{\mathbf{x}}$ direction

$$p_{\rm t} = \frac{1+\alpha_{\rm md} - \alpha_{\rm m}\alpha_{\rm d}}{(1-\alpha_{\rm m})(1-\alpha_{\rm d})} p_{\rm i}. \tag{6}$$

We can decompose the transmitted pressure into a bianisotropic pressure $p_{\rm b}$ due to the bianisotropic polarizability $\alpha_{\rm md}$ and a conventional pressure $p_{\rm c}$ due to the purely monopole-to-monopole $\alpha_{\rm m}$ and dipole-to-dipole $\alpha_{\rm d}$ metasurface responses. The bianisotropic and conventional pressures are obtained from equation (6) and are given by

$$\begin{aligned} p_{\rm b} &= \frac{\alpha_{\rm md}}{(1-\alpha_{\rm m})(1-\alpha_{\rm d})} p_{\rm i}, \\ p_{\rm c} &= \frac{1-\alpha_{\rm m}\alpha_{\rm d}}{(1-\alpha_{\rm m})(1-\alpha_{\rm d})} p_{\rm i}. \end{aligned} \tag{7}$$

Supplementary Note 2: Meta-atom Design

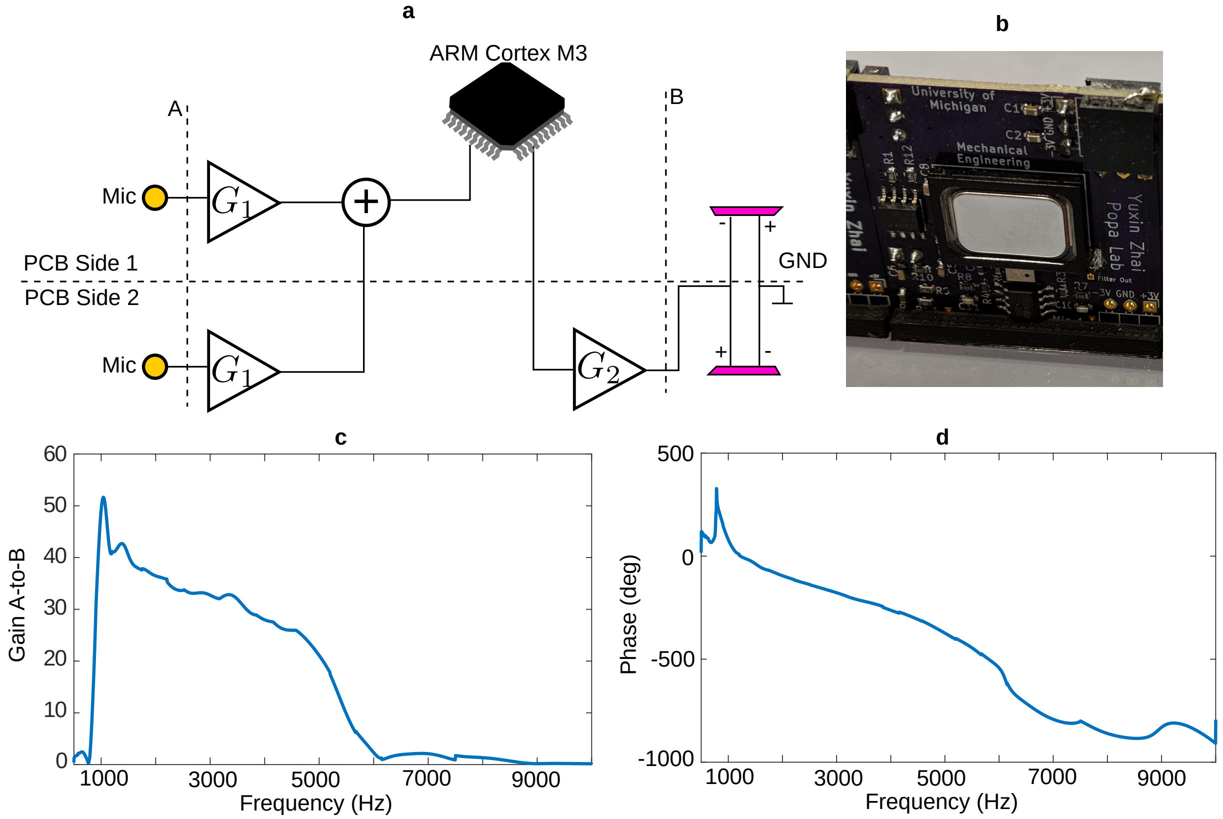

Supplementary Figure 2: Meta-atom design. **a**, Schematic of the meta-atom. **b**, Photo of fabricated bianisotropic meta-atom. **c**, The impulse response magnitude and d, phase of the meta-atom electronics between planes A and B.

The metasurface is composed of five meta-atoms. Each meta-atom is built on a 3.5 cm by 3.5 cm printed circuit board (PCB) and follows the sensor-driver architecture employed in other active metamaterials [8–10]. The meta-atom structure is shown in Supplementary Fig. 2a and Supplementary Figure 2b shows a close-up photo of the meta-atom. The bianisotropic response is engineered by employing a monopole transducer implemented via two MEMS microphones (Knowles SPQ0410HE5H) placed on each side of the PCB. The electrical signals produced by the two microphones are amplified using two non-inverting amplifiers of gain $G_1 = 67$ realized with the operational amplifier LM358 from STMicroelectronics and added in phase by an instrumentation

amplifier (INA132 from Texas Instruments). The resulting signal is sampled by an ARM Cortex M3 microprocessor. The microprocessor implements a digital infinite impulse response bandpass filter of pass band 500 Hz to 5000 Hz. The attenuation in the stop bands is 20 dB. The filtered signal drives a current amplifier of gain $G_2 = 0.5$ that provides the current needed by the driven transducers (AS01808AO produced by PUI Audio Inc). The transducers are placed on both sides of the PCB and driven 180° out-of-phase in order to generate a purely dipole moment. The electronics transfer function measured between planes A and B in Supplementary Fig. 2a is shown in Supplementary Fig. 2c. All the required electronics are mounted on the PCB except for the ARM Cortex M3 microprocessors. For increased flexibility each microprocessor is part of the commercial platform ArduinoDue and is connected to its meta-atom through three wires (input, output and ground). All unit cells are powered through 3 wires (±3 V and ground). The overall metasurface thickness including mounted components is approximately 9 mm.

Supplementary Note 3: Active Metasurface Stability

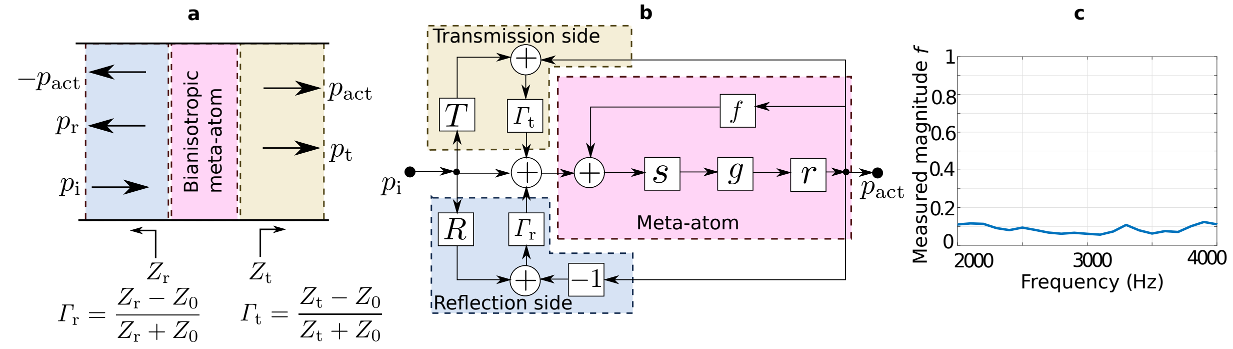

Supplementary Figure 3: Bianisotropic stability in the presence of external scatterers. **a**, The response of the bianisotropic meta-atom to the incident sound wave $p_{\rm i}$ is split into the passive transmitted $p_{\rm t}$ and reflected $p_{\rm r}$ waves and active bianisotropic response $p_{\rm act}$. The environment is modeled through effective impedances $Z_{\rm r}$ and $Z_{\rm t}$ looking towards the reflection and transmission sides; **b**, The flow graph describes the interaction between the incident wave and the wave scattered by the environment. The behavior of the sensor, active circuit, and driver are quantified by the impulse responses s, g, and r, respectively. The residual acoustic coupling between the driver and sensor caused by fabrication imperfections is quantified by the impulse response f. **c**, Measured f shows very low levels of residual coupling.

Active systems based on sensor-driver pairs have potential instability issues due to the feedback loops caused by 1) the close proximity of the sensor and driver (intra-meta-atom coupling), and 2) reflections of the sound produced by the dipole driver from the environment coupling back into the sensor (meta-atom-environment coupling). We show in this section that the active bianisotropic meta-atom is inherently stable even when embedded in complex environments and does not require any electronic feedback stabilization techniques that reduce the effectiveness of traditional active sound control techniques.

This remarkable stability comes from the requirement to realize the non-zero monopole-to-

dipole Willis coupling term. Namely, the meta-atom should sense the local pressure and generate a local velocity field that, in turn, has no contribution to the local pressure field. This design constraint translates into an active meta-atom in which the sensing and driven transducers are mechanically decoupled, i.e. the field produced by the driver is not sensed by the sensor. As shown in Supplementary Note 2, we realize this requirement by placing the pressure sensing transducer (i.e. monopole sensor) into the plane of anti-symmetry of the driven transducer (i.e. a dipole source). This geometry makes the dipole invisible to the sensing monopole, thus cancelling the intra-meta-atom feedback. Therefore, no additional circuitry is needed to stabilize the local feedback loop. This is in clear contrast with related approaches described in the literature in which active sound and vibration control performance and bandwidth are limited by stability constraints [11,12].

To show that the meta-atom is stable in the presence of external scatterers, we consider the test case of a meta-atom placed inside a one-dimensional waveguide. This scenario simulates an infinite metasurface (see Fig. 3a). We analyze the metasurface dynamic response to an incident wave of pressure p_{i} using the flow graph presented in Fig. 3b. Various components of the meta-atom and waveguide are represented through their impulse responses. Specifically, s, g, and r represent the impulse responses of the sensing, active, and driven elements respectively. The driver-sensor feedback path is modeled by the impulse response f, and the environment is modeled through the impedances Z_{r} and Z_{t} looking towards the wave source (reflection side) and away from the source (transmission side), or, equivalently, through the reflection coefficients Γ_{r} and Γ_{t} defined as shown in the figure in terms of Z_{r}, Z_{t}, and the background characteristic impedance Z_0. The passive reflection and transmission coefficients are defined as $R = p_{\mathrm{r}}/p_{\mathrm{i}}$ and $T = p_{\mathrm{t}}/p_{\mathrm{i}}$, respectively. From the block diagram we can express the transfer function $p_{\mathrm{act}}/p_{\mathrm{i}}$ and look at its stability requirements.

$$\frac{p_{\mathrm{act}}}{p_{\mathrm{i}}} = \frac{(1 + R\Gamma_{\mathrm{r}} + T\Gamma_{\mathrm{t}})sgr}{1 - (f - \Gamma_{\mathrm{r}} + \Gamma_{\mathrm{t}})sgr} \tag{8}$$

For maximum sound isolation we choose the active impulse response $g = -T/(sr)$ which leads to no transmission through the metasurface in the ideal case in which $f = 0$. With this choice of g, the Nyquist criterion becomes $|T||f - \Gamma_{\mathrm{r}} + \Gamma_{\mathrm{t}}| < 1$. Therefore, a sufficient condition for stability is $|f| < |T|^{-1} - |\Gamma_{\mathrm{r}}| - |\Gamma_{\mathrm{t}}|$.

Ideally, $f = 0$ but meta-atom fabrication imperfections makes f non-zero. To measure $|f|$, we performed two experiments. In both experiments we interrupt the direct path between g and r in Fig. 3b. The two on-board speakers are driven by a broadband chirp, e, having a Gaussian envelope and a 3 dB band of 2 kHz – 4kHz. The signal fed back to the front microphone, $s_1 = esrfg$, is recorded and compared against the signal received by the sensing monopole assembly when a third speaker, identical to the two on-board speakers, is placed 1 cm in front of the meta-atom and is driven by the same electrical signal e. The signal measured after the gain element g in the second experiment is $s_2 = esrg$. It follows that $f = s_1/s_2$. Figure 3c shows the measured $|f|$. The amplitude $|f|$ is highly sub-unitary in the octave 2 kHz – 4 kHz, and assures a stable meta-atom behavior for any passive environment.

Even in high gain media the meta-atom remains stable. For example, if the meta-atom is designed to have a passive transmission coefficient of magnitude $|T| \approx 0.1$ which is the scenario analyzed in this paper, it follows that the condition for stability becomes $|\Gamma_{\mathrm{r}}| + |\Gamma_{\mathrm{t}}| < 9.8$. In the most unfavorable scenario $|\Gamma_{\mathrm{r}}| = |\Gamma_{\mathrm{t}}| = 1$ and the stability condition is fulfilled. Remarkably, even in high gain media in which $|\Gamma_{\mathrm{r}}| > 1$ and/or $|\Gamma_{\mathrm{t}}| > 1$ the meta-atom remains stable as long as $|\Gamma_{\mathrm{r}}| + |\Gamma_{\mathrm{t}}| < |T|^{-1} - |f|$.

Supplementary References

1. Smith, D. R. Analytic expressions for the constitutive parameters of magnetoelectric metamaterials. *Phys. Rev. E* **81**, 036605 (2010).

2. Simovski, C. R. On electromagnetic characterization and homogenization of nanostructured metamaterials. *J. Optics* **13**, 013001 (2011).

3. Alù, A. First-principles homogenization theory for periodic metamaterials. *Phys. Rev. B* **84**, 075153 (2011).

4. Sieck, C. F., Alù, A. & Haberman, M. R. Origins of Willis coupling and acoustic bianisotropy in acoustic metamaterials through source-driven homogenization. *Phys. Rev. B* **96**, 104303 (2017).

5. Willis, J. R. Variational principles for dynamic problems for inhomogeneouselastic media. *Wave Motion* **3**, 1–11 (1981).

6. Milton, G. W., Briane, M. & Willis, J. R. On cloaking for elasticity and physical equations with a transformation invariant form. *New J. Phys.* **8**, 248 (2006).

7. Srivastava, A. Elastic metamaterials and dynamic homogenization: a review. *International Journal of Smart and Nano Materials* **6**, 41–60 (2015).

8. Popa, B.-I., Zigoneanu, L. & Cummer, S. A. Tunable active acoustic metamaterials. *Phys. Rev. B* **88**, 024303 (2013).

9. Popa, B.-I. & Cummer, S. A. Non-reciprocal and highly nonlinear active acoustic metamaterials. *Nat. Comms.* **5**, 3398 (2014).

10. Popa, B.-I., Shinde, D., Konneker, A. & Cummer, S. A. Active acoustic metamaterials reconfigurable in real time. *Phys. Rev. B* **91**, 220303 (2015).

11. Long, M. *Architectural Acoustics* (Elsevier Academic Press, Oxford, 2006).

12. Crocker, M. J. *Handbook of Noise and Vibration* (John Wiley and Sons Inc., New Jersey, 2007).
